# Vitality and Immunity

**Published:** 1903-02

**Authors:** Carl Schulin

**Affiliations:** Billings, Mont.


### VITALITY AND IMMUNITY.

By Carl Schulin, M.D.,
BILLINGS, MONT.

(Continued from page 6.)

Bacteriology is the natural history of the lowest plants on the borderland of the animal kingdom. It is a young but rapidly growing science and needs a Decandolle and a Darwin more than a Linné.

The most important feature of germs is their virulence. Their features are all more or less subject to variations. The virulence is the most variable feature. It must be caused by the production of certain chemical substances, either in the germs themselves or through their influence in the tissues. According as variations occur in the germs or the tissues, the results may be very different. The germs are much influenced by temperature, light, and other causes, and the tissues may at times contain substances which are missing at other times. The presence of other germs often plays an important rôle. This is called mixed infection.

We call a germ pathogenic if it is capable of producing substances in or on our bodies which are injurious to health. The complex of the symptoms produced constitutes a disease. If the same germ invades our bodies again and cannot reproduce the same disease, then we call ourselves immune against that disease. And this immunity is an acquired one. At the same time, we speak of congenital immunity in the case of germs which are injurious to some animals, but cannot do harm to others.

Immunity is known to exist not only against vegetable pathogenic germs, but in like manner against all kinds of animal parasites. They all favor certain animals and avoid others. This is partly caused by the relations between the different animals, which bring some into contact and keep others apart. Those in contact, for instance, dog and sheep, will naturally have some parasites in common. Still, no parasite can live on all animals, and no two kinds of animals which come regularly into contact have all their parasites in common. Individual immunity against almost any kind of parasite occurs frequently as well.

It seems to be the blessing of man that he has not only the greatest variety of animal parasites (according to Leuckart, about fifty kinds; as many as cattle and dogs together), but also the greatest variety of diseases. Man has congenital immunity only against a few diseases of minor importance; for instance, chicken cholera, swine septicæmia, swine plague, etc.; and, to make up for this loss, he has quite a number of diseases against which all animals are immune—*e. g.*, cholera, typhoid fever, lepra, scarlatina, gonorrhœa, and syphilis.

Animal parasites prefer some animals to others, probably for the same reason that plants select their habitat. It is a question of food. Seed reaches everywhere, but it cannot grow on sterile soil. This ought to apply to the pathogenic germs as well, and the animal parasites, too.

The leucocytes act similarly to those low-grade infusoria which are called amœbæ. They stretch out branches and thread-like twigs in all directions, apparently for the purpose of respiration and nutrition. In the presence of certain substances the movements become lively, while other substances have the opposite effect. This has been named positive and negative chemotaxis. Some germs have a positive chemotactic effect on the leucocytes. If capillary tubes containing pure cultures of such bacteria are introduced beneath the skin of animals, the leucocytes penetrate into the tubes. From other cultures the leucocytes keep away.

According to Buchner, it is an albuminous body in the bacteria which attracts the leucocytes. Gluten, casein from wheat, and legumin from peas have a similar effect while starch does not have it. The introduction of such substances into the blood produces a great increase in the number of leucocytes.

Absence of chemotactic force is identical with non-virulence as far as inflammation is concerned. And inflammation is one of the principal symptoms in most diseases concerning which we speak of immunity.

Chemotaxis is in many instances followed by phagocytosis (Metschnikoff)—*i. e.*, the taking up of germs into the bodies of leucocytes. Where more than one kind of germ is present the leucocytes sometimes reject a species which at other times they readily devour. Thus, in diphtheria combined with streptococcus infection, Ruffer found that the leucocytes took up the diphtheria bacilli only and not the cocci. In a mere streptococcus infection —*e. g.*, erysipelas—the leucocytes are not so particular. They devour the cocci, especially at the periphery, where the disease is spreading. In the older areas the cocci are nearly all free.

In some diseases, for instance smallpox and erysipelas, inflammation and fever are the most conspicuous symptoms and constitute nearly the whole disease. In others, for instance, tetanus and rabies, inflammation and fever play no rôles. Nervous symptoms are here in the foreground; other features present themselves in anthrax.

Anthrax is caused by an aërobic bacillus which grows superabundantly in the tissues and the blood. Its main symptom is an acute swelling, which leads not to suppuration, but to gangrene. Another characteristic symptom is the absence of fever. There may be a little fever in the beginning, but the temperature falls soon and becomes, especially in fatal cases, even subnormal. Death occurs under symptoms of irregular respiration and heart action, dilated pupils, diarrhœa, and bloody stools. These are symptoms of septicæmia. Hoffa isolated the poison which produces these symptoms from pure cultures of anthrax bacilli on sterilized meat. It is closely related to kreatin and is apparently not a product of the bacilli but a result of the decomposition of the meat by the germs growing on it.

The anthrax bacilli have negative chemotactic force. They drive the leucocytes away. Pus is formed only in favorably terminating cases when a line of demarcation establishes itself around necrotic parts. The wall of granulations stops the further progress of the bacilli and prevents reabsorption of septic material.

Anthrax bacilli eagerly consume oxygen. Therefore, Boellinger thought death was caused by asphyxia. In fatal cases, however, the oxygenating power of the blood has been found unimpaired (Nencki, in rabbits). Death is doubtless caused by poisons like the one which Hoffa found in meat, and the variations in different cases may depend upon differences in the chemical composition of the tissues on which the bacilli grow.

The anthrax bacillus was the first germ which was convicted of causing a disease. While it was seen as early as 1849 by Pollander, it was recognized as a pathogenic germ only in 1863 by Davaine, and in 1879 Toussaint demonstrated on the same germ for the first time the possibility of producing an artificial immunity against a disease. He made the discovery that animals which he injected with pure cultures of anthrax bacilli after they had been exposed to a higher temperature, were only slightly affected and remained thereafter immune. Pasteur and Koch confirmed this. After the anthrax bacilli, which thrive best at a temperature of 37° C., have been exposed for about a month to a temperature of 42° C., they are apparently unchanged, but no more poisonous. Koch succeeded in preserving such bacilli by continuous cultivation for more than two years.

After having been exposed for about nine days to a temperature of 42° C., the anthrax bacilli are still able to kill guinea-pigs, but no more rabbits. After two weeks more of such exposure to heat they can kill only mice, and later on not even these.

The anthrax bacillus does not produce an anthrax toxin in the same way the diphtheria bacillus produces a diphtheria toxin. Neither has there so far been found in the body an anthrax antitoxin. Still there is an immunity against anthrax. The anthrax bacillus can be so far attenuated that it can no more do any harm to the body, and, on the other side, the body can be fortified to such an extent that the strongest anthrax bacilli can no more injure it.

The acquired, as well as the congenital, immunity against anthrax has a peculiarity which, as far as we know, no other immunity has —it can easily be destroyed. White rats, dogs, fowls, frogs, also the Algerian race of sheep are naturally immune. The immunity of white rats, for instance, can be destroyed by compelling them to turn a wheel for a long time, and thus exhausting them (Roger). Artificial refrigeration does the same to fowls (Pasteur), inanition to pigeons (Canalis and Morpurgo). Curare, chloral, alcohol, etc., have the same effect. The immunity against anthrax which can be produced in man has no practical value. The time of incubation is too short to produce immunity after the infection has taken place.

Of the chemical composition of the diphtheria toxin as well as the antitoxin very little is known. The toxin is a very strong poison. It is a non-albuminous substance. In addition the diphtheria bacillus produces also a toxalbumin. The antitoxin is nonpoisonous to man and animals. It is an albuminous substance.

There is some difference of opinion concerning the relation between toxin and antitoxin. Some authors believe, with Buchner, that antitoxin is a chemically changed toxin, while others, with Ehrlich, accept it as an independent product of the animal body. The writer believes the latter correct, for the following reasons:

First, the just mentioned chemical difference between diphtheria toxin and antitoxin. A non-albuminous toxin could not very well change to an albuminous antitoxin.

Second. The quantitative proportion between toxin and antitoxin. This is not, for instance, like that between albumin and peptone during digestion, but it is about as the proportion between a regular dose of pilocarpine and the quantity of saliva secreted under its influence. Knorr found that a horse produced 100,000 times more tetanus antitoxin than toxin was furnished by the tetanus bacilli.

Third. The fact that a widespread opinion to the contrary notwithstanding, antitoxins do not protect exclusively against the toxins after which they are named. Ehrlich found that guinea-pigs can be made immune against the effects of the castor-oil bean and the jequirity bean by feeding them at first with small and then with gradually increasing doses of these beans. These immunities are caused by antitoxins which Ehrlich named antiricin and antiabrin, respectively. Antiabrin annuls the effects of snake venom and diphtheria toxin, as well as those of the jequirity bean; it protects also against ricin, but not against tetanus toxin. Tetanus antitoxin is antagonistic to snake venom, but not to abrin or ricin. Diphtheria antitoxin alone seems to protect against diphtheria toxin exclusively.

Some antitoxins are normal constituents of the blood of certain animals, while they are missing in others. This accounts for the natural immunity of certain animals against some diseases to which others are susceptible.

Antitoxins are used for the purpose of protecting the body by artificial immunization against infection. Providing the time of incubation be long enough, antitoxin may be used successfully even after the infection has taken place. Still, the field of this method is very limited in the great variety of diseases. The most splendid results are obtained with it in diphtheria.

Diphtheria antitoxin is a normal constituent of the blood of the horse. The horse cannot contract diphtheria, but the diphtheria toxin takes some effect on it. When injected hypodermically it causes some local irritation and a little fever. By slowly increasing the doses, the horse becomes more and more accustomed to the toxin, and its blood is charged with enormous quantities of diphtheria antitoxin. The serum of this blood can be used for the prevention and, if applied early enough, for the cure of diphtheria in man. Still, this artificial immunity is not lasting. That which is a normal constituent in the horse is in man a foreign element and will soon disappear from the blood, because it is not reproduced in the body. Infants are immune against diphtheria, and the disappearance of this immunity seems to coincide with the atrophy of the thymus gland.

Immunity, however, is not in every case produced by an antitoxin, as we already have observed in the case of anthrax. Nature, as a rule, does not have only one way of accomplishing a certain purpose.

Rabies has the longest, but the most varying, period of incubation. The average duration is six weeks; it varies, however, between a few days and twelve months. Basing his opinion on the results obtained with attenuated cultures of anthrax bacilli, Pasteur conceived the idea that the outbreak of rabies in a patient who had been bitten by a mad dog could be prevented in a similar way during the long period of incubation.

Unfortunately, all efforts to find the germ of rabies have so far been unsuccessful. Pasteur, however, although he could not isolate it, located it beyond doubt in the salivary glands, the pancreas, and the central nervous system. He also found that its virulence could be attenuated by drying pieces of the spinal cord of the diseased animal in a sterile atmosphere, the degree of attenuation being in proportion to the length of time it had been dry. He first succeeded in immunizing dogs, and later applied his famous treatment of rabies to man. At that time nothing was known of antitoxins, and therefore it is of great interest to compare Pasteur's views concerning rabies with our modern knowledge about an apparently similar disease—tetanus.

Both have in common the features that they originate from the infection of a lacerated wound and terminate fatally under convulsions from an affection of the central nervous system. The tetanus bacillus, however, in contradistinction to the supposed germ of rabies, is one of the best known of germs. It is an absolutely anaërobic bacillus. Outside of the body it leads a saprophytic life, apparently everywhere in the soil, especially where there is manure. It enters the body through lacerated wounds. Its toxin is one of the strongest poisons known. A mouse is killed by 0.00005 mg. This would make the lethal dose for a man about 0.23 mg., more than one hundred times less than that of strychnine.

The nature of the tetanus toxin is not fully understood. It is soluble in water, but does not diffuse through membrane. When taken into the mouth, it remains unabsorbed and is eliminated by the rectum (Ransom). In solution, as well as in a dry condition, it has an inclination to lose its poisonous properties. Acids and alkalies injure it rapidly; bile does the same. It is not an alkaloid; it is apparently a toxalbumin.

Congenital immunity against tetanus exists in amphibians. Birds are only slightly susceptible, most of them scarcely at all. Dogs are susceptible, but not so much as men, horses, mice, etc. Behring and Kitasato succeeded in producing artificial immunity by gradual introduction of toxin into the blood of different animals. They also obtained a well-characterized antitoxin. It was later on isolated from the blood of immunized dogs and prepared in a solid form by Tizzoni and Cattani.

An extremely interesting observation was made by Wassermann. He found that fresh guinea-pig brain or spinal cord rubbed up in a mortar with a normal salt solution and injected into animals produced immunity against tetanus lasting for twenty-four hours. Another equally interesting observation was made by Montesana and Montesson. They found a pure culture of attenuated tetanus bacilli in the cerebro-spinal fluid of a man who died from paralytic dementia without having had any symptoms of tetanus.

Two things are not kept sufficiently apart by writers on bacteriology, namely, the chemotactic force and the poisonous qualities of germs. The former is a physical phenomenon, while the latter have a chemical nature.

Manifold are the efforts to give a satisfactory explanation and definition of inflammation. The reader is familiar with the old neuropathic theory, with the theory of exsudation of the Vienna School; with Virchow's definition of inflammation as a phenomenon of irritation with the character of danger. A new era was inaugurated by Cohnheim's discovery of the active emigration of the leucocytes through the stomata between the endothelia. When bacteriology was developed, germs were charged with being the essential cause of inflammation. This, however, was soon found to be incorrect. Inflammation can be produced, without the presence of germs, by nitrate of silver, oil of turpentine, strong liquor of ammonia, and other substances, and the germs do not lose their power of producing inflammation after they have been killed by heat. Chemotactic force is not an active function of germs. It is a physical phenomenon, an electromagnetic attraction which certain substances exert on the leucocytes. All "activity" is on the part of the latter, and is, therefore, directly a function of the body. This function requires labor and material, such as only can be furnished by a healthy body. High-grade inflammation followed by the production of pus bonum et laudabile cannot take place in a deteriorated and vitiated system.

Concerning labor, the extremely interesting discovery has been made by Finsen that it is under the influence of the chemical rays of light. Finsen's assistant, Dr. Waldemar Vie, in an article prepared by request of Finsen and published in the *International Clinics*, vol. iii., eleventh series, 1901, describes the marvelous results obtained by him with his methods of phototherapy. Finsen found that in smallpox the suppuration in the vesicles can be prevented by excluding all but red rays of light from the sick room. In non-vaccinated people the "red-room treatment" has the same effect as vaccination when they contract smallpox.

Besides this, Finsen found that chronic and indolent inflammations can be cured with concentrated light, from which the heat rays have been excluded. The actinic rays produce not only inflammation, but, if applied in sufficient strength, they have a powerful germicidal action.

It appears from Finsen's discovery that the chemotactic force of germs is paralyzed when the actinic rays of light are excluded. Chemotaxis cannot take place in chemical darkness. On the other side, those incapsulated nodules with a slimy or cheesy centre, in which bacilli lead a saprophytic life, can be reached and disinfected with actinic rays of light.

Concerning the material of the inflammatory products, the discovery has recently been made, that if produced by a healthy body, they contain large quantities of the above-mentioned substance, from which the tissues obtain their antiseptic power.

Physicians of former ages, with their limited equipment for scientific research, assumed the existence of two things which play important rôles in pathology. One was the "materia peccans" of zymotic diseases and dyscrasia; the other was the vital force or "vitality" with which the system resists the materia peccans. The actual existence of the materia peccans was demonstrated by Pasteur and his school in the bacteria, and vitality was recognized by Brown-Séquard as a chemical substance produced by inner secretion in the body. In the last days of his life he made the discovery that the testicles contain this substance in larger quantities than any other part, and conceived the genial idea that senility might be treated successfully with injections of testicular extract. The name of the substance in question is spermin.

It is the great merit of Alexander Poehl, of St. Petersburg, Russia, to have investigated the spermin thoroughly, with the most gratifying results. The writer never in his life felt more pleased with a new medical work than when he read Poehl's "Spermin-theorie."

Spermin has its name from sperma, in which it was first discovered by Boettcher, in 1865. It has, however, nothing to do with fecundation. It does not produce or propagate life; it preserves it. It is the general antitoxin which the body produces indiscriminately against invading germs.

Spermin ($C_5H_{14}N_2$) is a strong base. With acids it forms salts which readily crystallize. Poehl uses for medicinal purposes hydrochlorate of spermin. It is readily soluble. Phosphate is under certain conditions insoluble, under others readily soluble. Such conditions are: the chemical reactions of the solvent. Perfect neutrality causes the phosphate of spermin to crystallize while an excess of acid, and especially of alkalinity, helps it to stay in solution. The presence or absence of certain other substances play a rôle. The so-called asthma crystals of Charcot and Leyden are insoluble phosphate of spermin and are identical with the crystals which Boettcher first obtained from sperma.

The best organs to obtain spermin from are the testicles of colts and stallions. Spermin, however, does not exist in these organs alone, but also in the ovaries, all glands, and especially in the hæmopoietic organs, spleen, bone-marrow, etc., and, besides, it is present in large quantities in healthy pus, in pus bonum et laudabile, but not in pus malignum!

Spermin has a catalytic force, that of transferring oxygen. Oxygen in statu nascendi is known to be a very strong antiseptic and thereupon is based the germicide force of the tissues and the blood.

Poehl established the following points: First, the power of promoting oxidation by the blood can be increased or impaired by the addition of certain substances. Spermin is one, by the presence of which this power is increased.

Second. By a lowering of the alkalinity of the blood, its power of promoting oxidation is impaired, and by the addition of spermin it is restored.

Third. Carbon oxide, chloroform, and other substances have a lowering influence in this regard, and are counteracted by spermin. Carbonate of soda has an accelerating influence on processes of oxidation, but it does not act so strongly as spermin. Chloride of sodium or ammonium, urea, allantoin, and leucin are indifferent. Urate of soda, certain leucomaines, and bile have a decreasing effect and are counteracted by spermin.

Fourth. By the injection of urine into the blood the power of oxidation is greatly diminished, especially by the urine of anæmic people, of people who have neoplasma ventriculi, spondylitis, ischias, and of those who are under the influence of shock. This can also be counteracted by the introduction of spermin into the blood.

Fifth. The *modus operandi* of spermin has a catalytic character. It is itself not changed by its work. It can, however, work only as long as it is in an easily soluble condition, as in the case of hydrochlorate. Phosphate of spermin, which is difficult of solution, is inert.

Poehl, then, calls the attention of his readers to the so-called "textural respiration" and the products of retrogressive metamorphosis in the tissues. Of the latter, especially those which Gautier placed together as "leucomaines," play an important rôle in pathology.

The albuminous substances are mixtures of chemical compounds which resemble one another. They may be homologous. So far we are not enabled to separate these substances sharply from one another. Attempts have been made to gain an insight into their chemical constitution from the products of their decomposition in the metabolism. Here we must never forget that the "giant molecules" with which we have to deal are not satisfied with one chemical action. At one end of their bodies they may be an acid, and at the same time at another end a base; at a third end may be an aldehyde, etc., and they always play several rôles simultaneously.

The reader has observed that even spermin, which is a comparatively plain chemical compound, is at the same time a base and an enzyme.

A good many processes of life go on without disintegration of the albuminous molecule. The power is apparently furnished by means of some physical agency, especially the actinic rays of light. A good many other processes, however, involve the decay of the molecule and lead to the formation of a great number of products of splitting. Two chemical actions stand here in the front rank, that of oxidation and that of hydration of parts of the giant molecule.

The first changes from retrogressive metamorphosis are so slight that they are hardly recognizable. Still, they are sometimes sufficient to impart the strongest toxic qualities. Therefore, these albumins have been named toxalbumins in contradistinction to the original ones. Poehl thinks he found in polarized light the means of detecting the very first beginning of these changes; in a similar way as it is known from grape sugar.

In the further stages of the retrogressive metamorphosis, substances form which are chemically different from the original albumins. Of these substances, those which contain nitrogen, the leucomaines, especially deserve our attention. They have the feature in common that they can easily be oxidized and changed to other substances which are more readily soluble and diffuse more easily. Those leucomaines which are poisonous lose their toxicity by oxidation.

Oxidation thus performs the important function of changing products of imperfect retrogressive metamorphosis in such a way that they can more easily be eliminated and lose their objectionable qualities. To do harm, these products need not absolutely be poisonous. Uric acid, for instance, is not a poison, but it causes trouble mechanically by its accumulation in the tissues. Uric acid, which is barely soluble, can, by oxidation, be changed to urea, which is readily soluble, and is, therefore, easily eliminated.

The accumulation of such substances in the blood and tissues is the essential feature of those diseases which Bouchard put together under the head of auto-intoxications.

Albumin contains nitrogen in two forms. Part of it is united with the oxygen-containing radicals CO and CO–CO. These radicals give the molecules the feature of amido compounds. Here belong urea and the ureides. The balance of the nitrogen is united with hydrocarbon radicals. They impart to the nitrogen its basic qualities. The narrow limits of this paper do not permit of the enormous number of substances which are thus produced being enumerated. Suffice it to state, however, there are four groups of them: the ureides, those of the xanthin group, those of the kreatin group, and the neurin bases.

Of the ureides, the most important is uric acid. Of other members of this group, only two may be mentioned, allantoin and alloxan, which need no further introduction to the reader. All ureides can easily be changed to urea, directly or indirectly by oxidation or hydration, respectively. No ureide is poisonous.

The members of the xanthin group all have more or less the features of alkaloids and are at the same time bases and weak acids. According to Kossel, they are all the splitting products of nuclein and nucleinic acid, respectively. From the nucleinic acid of yeast Kossel gained guanin and adenin; from that of the testicles adenin, xanthin and sarcin; from that of the thymus gland adenin.

The xanthin compounds are found in all glands. In the opinion of Kossel, they originate from the nucleinic acid in the nuclei of the cells in the glands. By far the greatest part of them is by oxidation changed to uric acid. A very small amount is eliminated unchanged by the kidneys. Adenin ($C_5H_5N_5$) has been found in all glands, but never in the muscles. Adenin, by oxidation, is changed to alloxan. It is not present in normal urine, but has been found in the urine of people with leukæmia, also of children with nephritis.

Xanthin and sarcin, which is also called hypoxanthin, are found in all tissues which are rich in nucleinic acid. They are also found in muscles. Traces of both are normal constituents of the urine, and they are found in increased quantities in the urine of people with leukæmia and nervous diseases. Both are toxic; sarcin more than xanthin. They produce muscle spasm and later paralysis of the spinal cord. They also irritate the kidneys. Guanin is not toxic. Like xanthin and sarcin, it can, by oxidation, be changed to uric acid.

The members of the kreatin group, kreatin, kreatinin, etc., are all found in large quantities in the muscles. By oxidation through some intermediary stages, they produce urea—not uric acid! They are toxic; they produce convulsions. In very small quantities they are normal constituents of the urine. These quantities are much increased in disease. While the members of the xanthin group are splitting-products of the nucleo-albumin, those of the kreatin group originate in the protoplasma, which accounts for their prevalence in the muscles. The muscles perform their functions pre-eminently with protoplasma, while in other parts, for instance, the glands and the hæmopoietic organs, metabolism of the nuclei prevails.

(To be continued.)

